# CZK, a novel alkaloid derivative from *Clausena lansium*, alleviates ischemic stroke injury through Nrf2-mediated antioxidant effects

**DOI:** 10.1038/s41598-023-32999-2

**Published:** 2023-04-13

**Authors:** Haodong Chen, Yangbo Liu, Juling Feng, Hongyun Wang, Yantao Yang, Qidi Ai, Zhao Zhang, Shifeng Chu, Naihong Chen

**Affiliations:** 1 Hunan Engineering Technology Center of Standardization and Function of Chinese Herbal Decoction Pieces & College of Pharmacy, Hunan University of Chinese Medicine, Changsha, 410208 China; 2 State Key Laboratory of Bioactive Substances and Functions of Natural Medicines, Institute of Materia Medica & Neuroscience Center, Chinese Academy of Medical Sciences and Peking Union Medical College, Xian Nong Tan Street, Beijing, 100050 China

**Keywords:** Stroke, Network topology, Pharmacodynamics, Neurological models

## Abstract

Anti-oxidant stress is a potential strategy for the treatment of ischemic stroke. Here, we found a novel free radical scavenger termed as CZK, which is derived from alkaloids contained in *Clausena lansium*. In this study, we first compared cytotoxicity and biological activity between CZK and its parent’s compound Claulansine F. It was found that CZK had lower cytotoxicity and improved anti-oxygen–glucose deprivation/reoxygenation (OGD/R) injury than its parent’s compound. Free radical scavenging test showed that CZK had a strong inhibitory effect on hydroxyl free radicals with the IC_50_ of 77.08 nM. Intravenous injection of CZK (50 mg/kg) significantly alleviated ischemia–reperfusion injury, manifested by reduced neuronal damage and decreased oxidative stress. Consistent with the findings, the activities of superoxide dismutase (SOD) and reduced glutathione (GSH) were increased. Molecular docking predicted that CZK might be combined with nuclear factor erythroid 2-related factor 2 (Nrf2) complex. Our results also confirmed that CZK upregulated the contents of Nrf2 and its target gene products Heme Oxygenase-1 (HO-1), and NAD(P)H: Quinone Oxidoreductase 1 (NQO1). In conclusion, CZK had a potential therapeutic effect for ischemic stroke by activating Nrf2-mediated antioxidant system.

## Introduction

Ischemic stroke, which accounts for approximately 87% of all strokes, is a leading cause of disability and death worldwide. The main cause of ischemic stroke is cerebral vascular embolism, and its treatment focuses on vascular recanalization^1^. Recombinant tissue plasminogen activator (rt-PA) is the foremost measure to restore blood flow^2^. However, the therapeutic time window of rt-PA is only 4.5 − 6 h, and inappropriate treatment may cause intracranial hemorrhage^3^. Therefore, there is still an urgent need to develop novel agents for the treatment of ischemic stroke.

Oxidative stress plays a significant role in the pathogenesis of ischemic stroke, manifested as the accumulation of reactive oxygen species (ROS). The excessive production of ROS results in the exhaustion of oxidoreductase and the redox imbalance, which leads to oxidative stress injury and ischemic cascade reactions such as energy metabolism disorder, inflammation, and apoptosis^4,5^. To maintain optimal redox balance in our body, it is also necessary to improve the oxidative defense system to resist oxidative stress damage caused by various stimulates. As a key transcriptional factor of anti-oxidative genes, nuclear factor erythroid 2-related factor 2 (Nrf2) has ability to regulate hundreds of anti-oxidative and anti-inflammation genes production through binding to the antioxidant response element (ARE)^6^. Hence, removing excess ROS and promoting Nrf2-related signaling pathways are beneficial to improve oxidative stress after ischemic stroke. Free radical scavenger is a kind of neuroprotective agent capable of removing excessive ROS. Edaravone, a classical free radical scavenger, is extensively used in China, Japan, and South Korea for the treatment of ischemic stroke. It could scavenge hydroxyl radicals to protect neurons against oxidative stress and improve the tolerance of brain tissue to hypoxia and ischemia^7^. Other neuroprotective agents such as NXY-059 showed good therapeutic effects in ischemic stroke animals. However, there still exist some problems with clinical transformation^8^. It is urgent to find more potential and safe free radical scavengers as drug candidates for ischemic stroke.

*Clausena lansium* (Lour.) Skeels, also known as wampee, belongs to the Rutaceae family which is distributed in south China. Previous studies have demonstrated that the bioactive compounds of *Clausena lansium* have hepatoprotective activity, anti-inflammation, anti-oxidation, anti-cancer, and neuroprotection^9–12^. Claulansine F, a carbazole alkaloid isolated from the stem of *Clausena lansium*, could be used not only as an anti-Alzheimer’s Disease agent but also to improve ischemic stroke^13,14^. Claulansine F had a powerful free radical scavenging ability and significantly inhibited the production of ROS in vivo. However, there is an aldehyde group in the structure of Claulansine F, which may lead to a high risk of cytotoxicity. To improve its security, a series of CZ derivatives designed by Claulansine F were studied (patent number: CN201611213048.6). In these compounds, the neuroprotective effect of CZ-7 was better than that of Claulansine F, and it had therapeutic potential for ischemic stroke, vascular dementia, and demyelinating disease^14–16^. However, CZ-7 had low solubility and druggability, so we designed a new derivative called CZK. Whether CZK still plays a role in ischemic stroke and the specific therapeutic mechanism needs to be further clarified. We used the molecular docking and rat middle cerebral artery occlusion (MCAO) model in this study to explore the mechanism of CZK treating ischemic stroke.

## Material and methods

### Material

The compound CZK (purity > 98% by HPLC analysis, molecular formula: C_24_H_24_KNO_3_; molecular weight: 413) and Claulansine F (purity > 98% by HPLC analysis, molecular formula: C_19_H_17_NO_3_; molecular weight: 307) were supplied by the Institute of Materia Medica, Chinese Academy of Medical Sciences & Peking Union Medical College (Beijing, China). Edaravone injection was purchased from Simcere (Nanjing, China). Dulbecco’s modified eagle medium (DMEM) was bought from TransGen Biotech (Beijing, China). Fetal bovine serum was acquired from Thermo Fisher (MA, USA). CCK-8 kit was provided by TargetMOI (Shanghai, China). Dihydroethidium (DHE, D7008) and 2,3,5-triphenyltetrazolium-chloride (TTC, T8877) were obtained from Sigma-Aldrich (St. Louis, MO, USA). The primary antibodies Nrf2 (ab62352, 1:1000 dilution; ab31163, 1:200 dilution) and 8-hydroxy-2’-deoxyguanosine (8-OHdG, ab62623, 1:100 dilution) were acquired by Abcam (Cambridge, UK); β-actin (AC038, 1:10,000 dilution) and NAD(P)H: Quinone Oxidoreductase 1 (NQO1, A1518, 1:1000 dilution) were acquired by Abclonal (Wuhan, China); Kelch-like ECH-associated protein 1 (Keap1, 8047S, 1:1000 dilution) and Heme Oxygenase-1 (HO-1, 82206S, 1:1000 dilution) were acquired by Cell Signaling Technology (Danvers, MA, USA).

### Cell culture and cytotoxicity comparison

The human neuroblastoma SH-SY5Y cells were cultured in DMEM containing 10% fetal bovine serum and incubated with a humid atmosphere of 5% CO_2_ at 37 ℃. For the cytotoxicity test, SY5Y cells (1.5 × 10^4^ cells/mL) were implanted into a 96-well plate and allowed to adhere to the wall. Then the medium was replaced with DMEM containing CZK or Claulansine F, and the cells were continuedly cultured for 24 h, 48 h, or 72 h.

### Establishment of oxygen–glucose deprivation/reoxygenation (OGD/R) model

An OGD/R model was established to simulate ischemia–reperfusion. In short, SH-SY5Y cells were seeded as described above. Then the medium was replaced with glucose-free Earle’s solution, and the cells were placed in a hypoxia incubator chamber and continuously injected with 99.999% N_2_. After lacking oxygen and glucose for 5 h, Earle’s solution was removed and replaced with the complete or medicated medium. The cells were put into the incubator for 24 h for reoxygenation.

### Cellular viability assay

The CCK-8 kit was used for detecting cell viability. After the culturing, 10 μL of CCK-8 working solution was added to the cells and incubated at 37 °C for 2 h. The absorbance at 450 nm was measured by a multimode microplate reader (Thermo Fisher, MA, USA).

### Detection of free radical scavenging capability of CZK

Electron Paramagnetic Resonance (EPR) is an important method to detect free radicals. Briefly, hydroxyl radical was produced via the Fenton reaction^17^. CZK was dissolved in dimethyl sulphoxide and put into a quartz tube, then they were quickly inserted into the EPR resonator. The EPR spectra were recorded at 25 °C with a Bruker EPR spectrometer (MA, USA). Measuring parameters were set as: center field: 3510 G; sweep width: 100 G; frequency: 9.85 GHz; modulation amplitude: 1 G; modulation frequency: 100 kHz; conversion time: 45 ms.

### Animals

Male adult SD rats (about 8 weeks, 250 − 280 g) were purchased from Charles River Laboratories (Beijing, China). All rats were housed under the following conditions: temperature: 23 ± 1 °C, humidity: 50 ± 5%, and 12 h day/night cycle. Rats were acclimatized for 3 days before the operation and provided with adequate food and water during the experiment. They were euthanized with isoflurane at the end of neurobehavioral test. All experiments were approved by Animal Care and Use Committee of the Peking Union Medical College and Chinese Academy of Medical Sciences (Ethical inspection No. 00003907). The study was performed with the protocols in the NIH Guide for the Care and Use of Laboratory Animals and carried out in compliance with the ARRIVE guidelines^18^.

### MCAO and drug administration

Rats were randomly divided into 6 groups: Sham, MCAO, Eda, CZK low-dose, CZK medium-dose, and CZK high-dose. The MCAO model used in this study was operated as previously described^19^. Briefly, rats were allowed to water but no food for 12 h before MCAO surgery. They were anesthetized with 3% isoflurane, then the right common carotid artery (CCA), external carotid artery (ECA), and internal carotid artery (ICA) were exposed. A silicone rubber coated monofilament (L3800, Guangzhou Jialing Biotechnology Co., Ltd., Guangzhou, China) was inserted from the ECA into the ICA and then occluded the middle cerebral artery (MCA). After 90 min of occlusion, the monofilament was pulled out to allow reperfusion. Sham-operated rats received the same procedures without MCA occlusion.

Rats were administered intravenously during the beginning of reperfusion: Sham and MCAO groups were given saline; Eda group was given Edaravone injection (10 mg/kg); CZK low-dose, medium-dose, and high-dose groups were given CZK solution (10 mg/kg, 25 mg/kg, 50 mg/kg) respectively.

### Neurological deficits test

Zea longa test was performed to evaluate the neurological deficits as previously described^20^. Scoring criteria are as follows: 0 points, no neurological defect; 1 point, unable to entirely stretch the left forelimb; 2 points, toward the body to the left while walking; 3 points, dump to the left while standing; 4 points, unable to walk spontaneously and lose consciousness; 5 points, death. The rats with 0 or 5 points were excluded from the statistics.

### TTC staining

TTC staining was used for the assessment of the infarct areas. After reperfusion of 24 h, rats were anesthetized with 3% isoflurane and sacrificed, and the brains were quickly collected. Brains were cut into six consecutive coronal slices (2 mm) and stained with 2% TTC solution at 37 °C for 15 min, and then slices were fixed in 4% paraformaldehyde overnight. The infarct area and edema volume were analyzed using ImageJ v1.6.0 software: infarct ratio (%) = total infarct area/(2 × contralateral brain area) × 100%; edema ratio (%) = ipsilateral volume/(2 × contralateral volume) × 100%.

### Nissl staining

Nissl staining was used to observe neuronal damage after brain ischemia–reperfusion. The brain slices were treated with toluidine blue for 5 min, then rinsed with pure water and decolorized in xylene for 10 min. After processing, the slices were mounted with neutral gum. Nissl-stained cells in the cortex and the striatum area were observed under a light microscope (Olympus, Tokyo, Japan).

### Detection of antioxidants and oxidation products

Superoxide Dismutase (SOD) assay kit (A001-3) and Malondialdehyde (MDA) assay kit (A003-1) were acquired by the Nanjing Jiancheng Institute of Biological Engineering (Nanjing, China). Reduced glutathione (GSH) and oxidized glutathione disulfide (GSSG) assay kit (S0053) was purchased from Beyotime Biotechnology (Shanghai, China). SOD, MDA, and GSH/GSSG levels were detected according to the manufacturer’s instructions. Before the test, the brain tissues were weighed and homogenized with normal saline at 1:9 proportion. The supernatants were collected by centrifuging at 3000 rpm for 10 min and used as samples.

### DHE staining

DHE would be oxidized by intracellular ROS to produce red fluorescence, which could represent the change in ROS content. Rats were perfused with Phosphate Buffered Saline (PBS) followed by 4% paraformaldehyde, and the brains were quickly collected and made into frozen slices (20 μm). The slices were boiled for antigen retrieval for 10 min. Then, they were recovered to 25 °C and washed 3 times with PBS. Next, they were permeabilized by 0.5% (v/v) Triton X-100 in PBS for 10 min and washed 3 times with PBS. Finally, the slices were incubated with DHE (100 μM) in the dark at 37 °C for 2 h. Slices were filmed through a confocal laser scanning microscope (Leica, Solms, Germany).

### Molecular docking

For ligand preparation, 3D chemical structure was created and energy was minimized for the chosen ingredient by ChemBioDraw 3D software. For receptor, the crystal structure of Keap1-Nrf2 (ID: 4XMB) was downloaded from the RCSB Protein Data Bank (PDB) database and modified using Discovery Studio v16.1.0 software to remove ligands and water, add hydrogen, and optimize and patch amino acids. Next, the ligand was docked into the prepared receptor by the “Receptor-Ligand Interactions” module. After the completion of docking, the model with a higher score was selected for analysis to examine the types of interactions.

### Western blot

Western blot was performed as previously described^21^. Ischemic penumbras were separated and lysed at 4 °C for 30 min. The supernatants were collected by centrifuging at 12,000 rpm for 30 min, and BCA assay was applied to measure protein concentrations. Each protein sample was adjusted to equal amount and subjected to 10% (w/v) SDS-PAGE gels, following which they were transferred to PVDF membranes. After that, the membranes were blocked with 5% (w/v) bovine serum albumin (BSA). Then the membranes were cut according to the target protein molecular weight and blotted with the following primary antibodies overnight at 4 °C: Nrf2, Keap1, NQO1, HO-1, β-actin, then incubated with horseradish peroxidase-conjugated secondary antibodies at 25 °C for 2 h. ECL plus detection system (Molecular Device, Lmax) was used for assaying the content of each protein. The intensities of bands were quantified by ImageJ v1.6.0 software.

### Immunofluorescence

The brain slices were prepared as previously described. Antigen-repaired slices were blocked with 5% (w/v) BSA for 30 min, slices were incubated with Nrf2 and 8-OHdG antibodies at 4 °C overnight. The next day, slices were washed with PBS-T for 3 times and then incubated with secondary antibodies at 25 °C for 2 h. The slices were filmed through a confocal laser scanning microscope.

### Statistical analysis

All statistical analyses were processed with GraphPad Prism v8.4.3 software, and results were presented as means ± SEM. Comparisons between compounds in cytotoxicity were performed in Student’s *t* test. Significance in the remaining experiments was determined by one-way ANOVA followed by Dunnett’s test. Details of the statistical analysis are indicated in the figure legends, and *p* < 0.05 was considered as the standard of statistical difference.

## Result

### Cytotoxicity and neuroprotective effects of CZK

**Figure 1:**
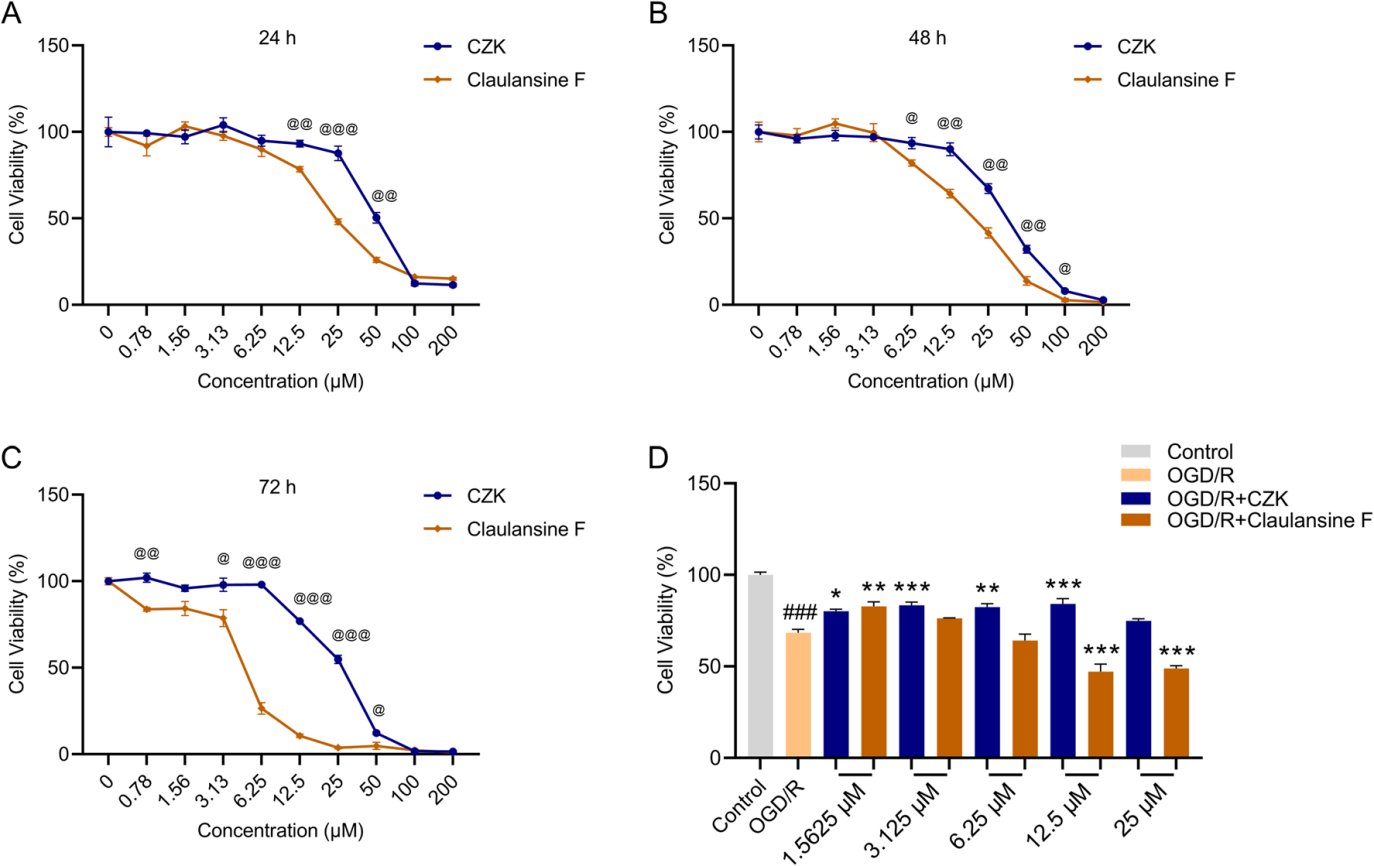
Cytotoxicity and neuroprotective activity of CZK in vitro. Cytotoxicity of CZK and Claulansine F in (**A**) 24 h, (**B**) 48 h, and (**C**) 72 h. (**D**) Neuroprotective activity of CZK and Claulansine F in OGD/R-induced SH-SY5Y cell injury. Data were presented as the mean ± SEM (*n* = 3). ^@^*p* < 0.05, ^@@^*p* < 0.01, ^@@@^*p* < 0.001 vs. Claulansine F group. ^###^*p* < 0.001 vs. Control group. ^*^p < 0.05, ^**^p < 0.01, ^***^p < 0.001 vs. OGD/R group. For cytotoxicity, Student’s *t* test. For OGD/R, one-way ANOVA, interaction, *F* (11, 24) = 45.39, *p* < 0.0001.

Toxicity of molecules is a very important part of drug development. CZK was created to reduce the potential toxicity of Claulansine F. For this reason, we predicted the biotoxicity of CZK and Claulansine F by ADMETlab and ProTox-II database^22,23^. It indicated that Claulansine F might produce carcinogenicity, mutagenicity, and AMES toxicity. And the modified CZK showed no above toxicity (Table 1). In addition, we used the SH-SY5Y cells to investigate the cytotoxicity of CZK and its parent’s compound Claulansine F. As shown in Fig. 1A, when incubated with the drugs for 24 h, CZK and Claulansine F showed no cytotoxicity at a concentration below 12.5 μM. However, with the extension of incubation time, especially at 72 h, the cell viability of Claulansine F was significantly lower than that of CZK at the concentration of 6.25 μM (Fig. 1B,C).

**Table 1:** Prediction of toxicities.

	Claulansine F	CZK	Source	Website
hERG blocker	Yes	No	ADMETlab	https://admet.scbdd.com/home/index/
Hepatotoxicity	No	No		
AMES mutagenicity	Yes	No		
Skin sensitization	No	No		
Carcinogenicity	Active	Inactive	ProTox-II	https://tox-new.charite.de/protox_II/
Immunotoxicity	Active	Active		
LD_50_	522 mg/kg	850 mg/kg

Furthermore, we determined the neuroprotective effects on cell injury induced by OGD/R. It was shown that CZK at the concentration from 1.5625 to 12.5 μM could reverse the damaging effect. Unlike CZK, Claulansine F only had a protective effect at 1.5625 μM, once beyond this concentration range, Claulansine F would lose the therapeutic effect and even produce acute cytotoxicity (Fig. 1D). The above results showed that CZK had lower cytotoxicity than Claulansine F.

### CZK had an efficient free radical scavenging ability

**Figure 2:**
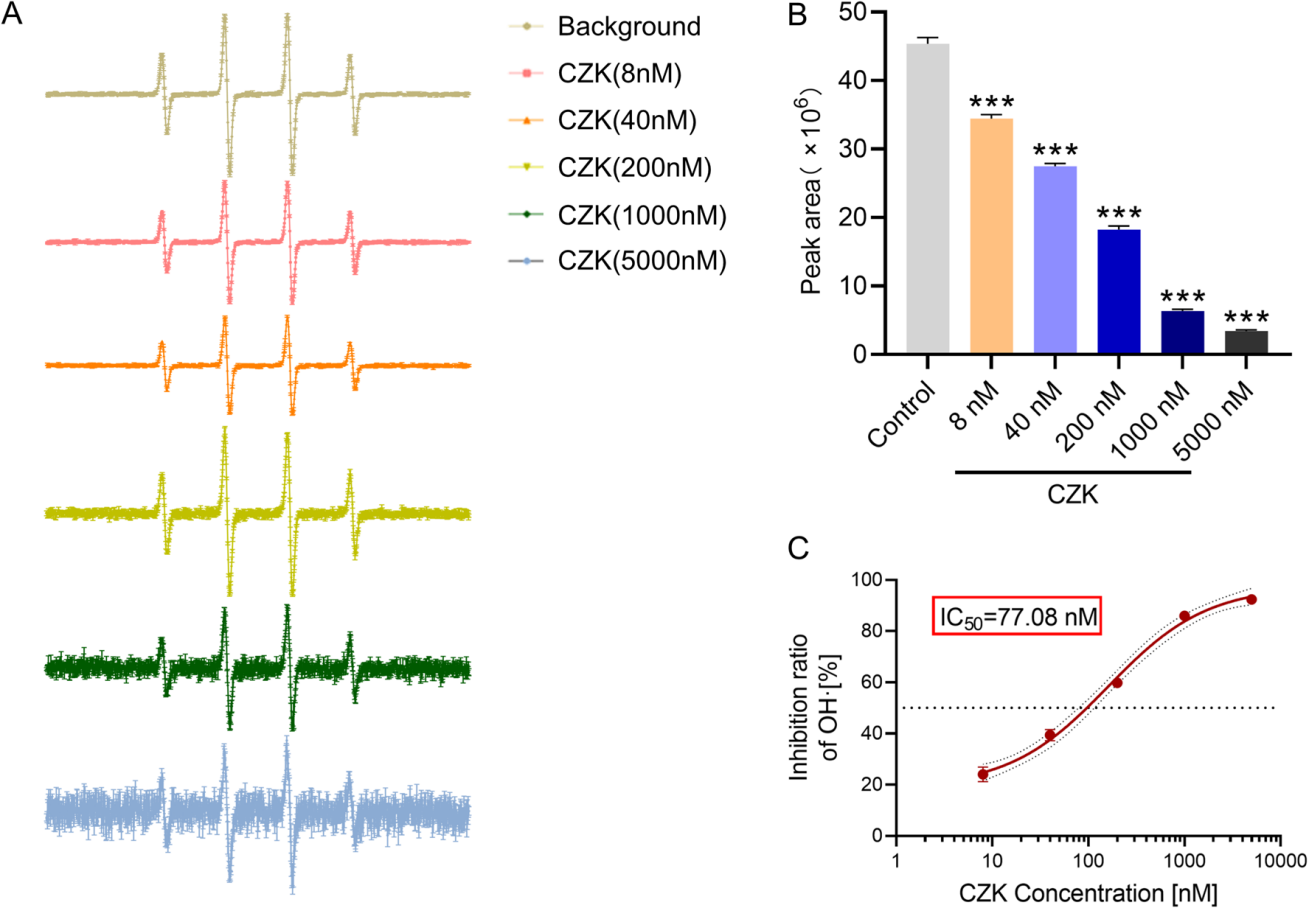
Effect of CZK on the production of hydroxyl radicals in Fenton reaction system. (**A**) The intensity of OH· scavenging. (**B**) Peak area analysis of hydroxyl radicals scavenging by different concentrations of CZK. (**C**) Half maximal inhibitory concentration. IC_50_ = 77.08 nM. Data were presented as the mean ± SEM (*n* = 3). ^***^p < 0.001 vs. Control group. For peak area, one-way ANOVA, *F* (5, 12) = 2609, *p* < 0.0001.

Hydroxyl radical is one of the most common ROS groups in the body, which can react with almost all biological macromolecules, and the reaction speed is fast and aggressive^24^. Hence, we chose hydroxyl radical as the detection index to test the scavenging ability of CZK. In this test, CZK had a strong scavenging ability for hydroxyl radicals. With the increase in concentration from 8 to 5000 nM, it showed a dose-dependence manner (Fig. 2A,B) to decrease the content of hydroxyl radical. According to the calculation, the IC_50_ was equal to 77.08 nM (Fig. 2C). In our previous research, we tested the hydroxyl radical scavenging ability of Edaravone, and IC_50_ was equal to 790 nM^14^. Through the comparison between CZK and Edaravone, we found that the hydroxyl radical scavenging ability of CZK was much stronger than that of Edaravone.

### CZK alleviated ischemia–reperfusion-induced infarction and neurological deficits in rats

**Figure 3:**
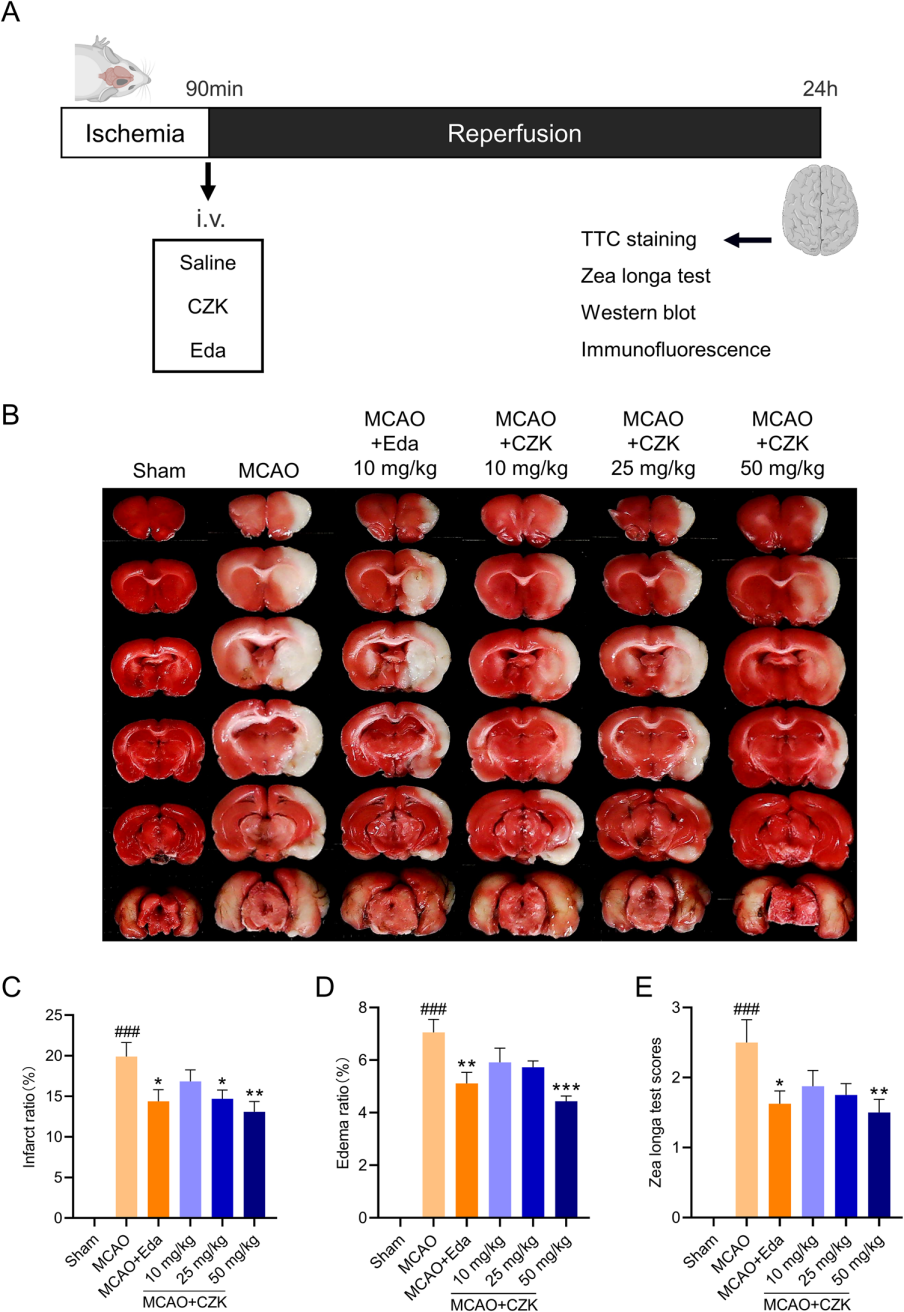
CZK protects against ischemic stroke-induced brain injury. (**A**) The scheme of the experiment. (**B**) Representative image of TTC staining 24 h after 24 h of reperfusion. (**C**,**D**) Infarct and edema ratio. (**E**) Statistical analysis of the Zea Longa scores. Data were presented as the mean ± SEM (*n* = 8). ^###^*p* < 0.001 vs. Sham group. ^*^p < 0.05, ^**^p < 0.01, ^***^p < 0.001 vs. MCAO group. For infarct ratio, one-way ANOVA, *F* (5, 42) = 28.49, *p* < 0.0001. For edema ratio, one-way ANOVA, *F* (5, 42) = 45.28, *p* < 0.0001. For Zea longa scores, one-way ANOVA, *F* (5, 42) = 16.31, *p* < 0.0001.

As a derivative of Claulansine F, the therapeutic effect of CZK on ischemic stroke is not clear. Here, we used MCAO models to evaluate the dose-dependent response of CZK in the treatment of ischemic stroke from 10 to 50 mg/kg (Fig. 3).

TTC staining exhibited that the infarct area in the CZK group was decreased with the increase of CZK’s dosage, which showed significant differences at the dose of 25 and 50 mg/kg (Fig. 3B,C). When the dosage reached to 50 mg/kg, the edema ratio analysis and Zea longa test presented similar downward trends as the infract ratio decreased, which showed a significant difference when compared to that in MCAO group (Fig. 3D,E). All of these results suggested that CZK could protect against ischemic stroke, and 50 mg/kg had the optimal therapeutical effect. Therefore, 50 mg/kg was used as the therapeutic dose in the follow-up experiments.

### CZK reduced oxidative stress injury after cerebral ischemia–reperfusion

**Figure 4:**
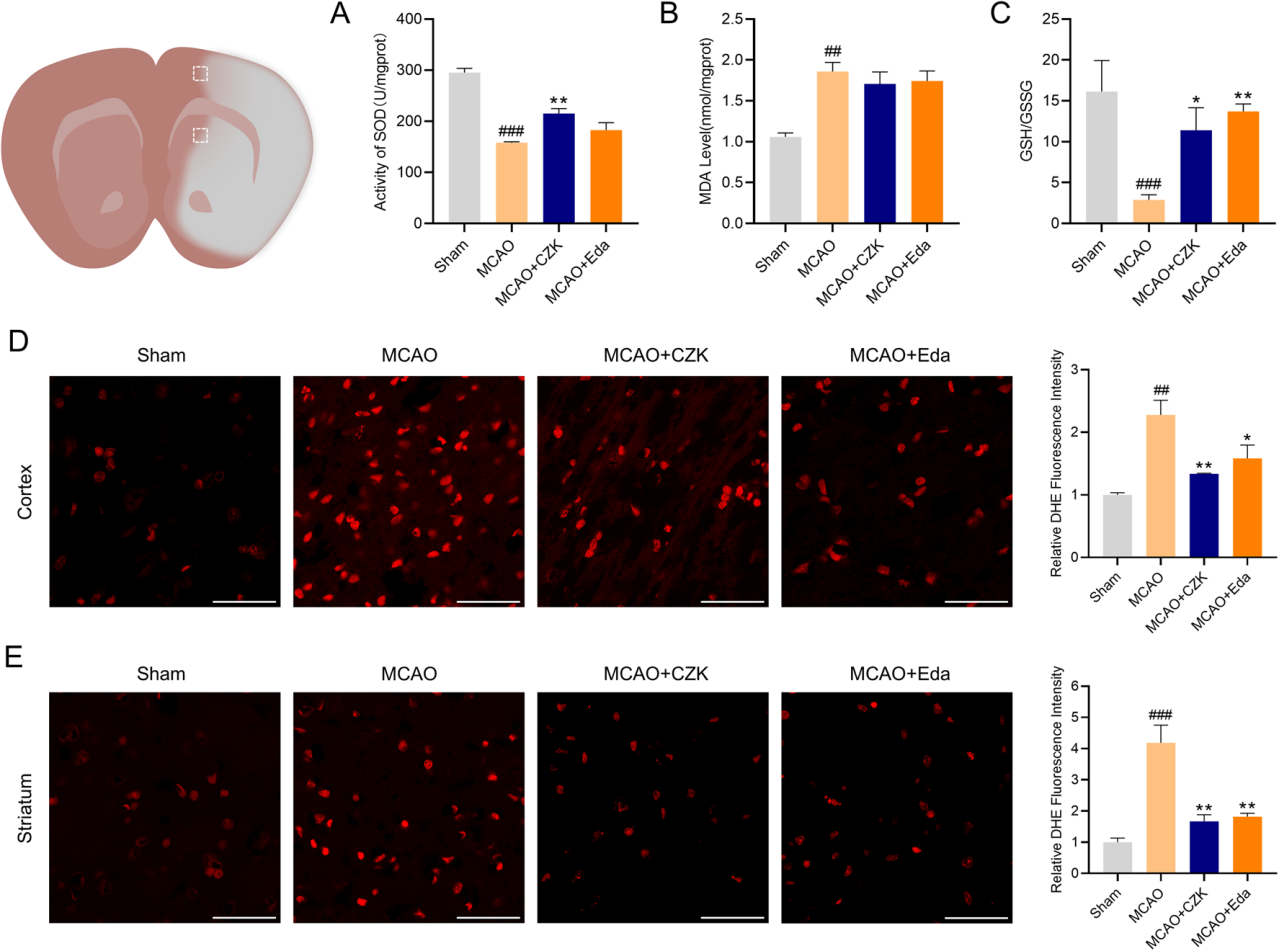
CZK reduced oxidative stress levels at 24 h after MCAO. (**A** − **C**) The effects of CZK on the activity of SOD, MDA level, and GSH/GSSG. (**D**) Representative microphotograph of DHE staining in the cortex region and quantification of fluorescence intensity. (**E**) Representative microphotograph of DHE staining in the striatum region and quantification of fluorescence intensity. Scale bar = 50 μm. Data were presented as the mean ± SEM (*n* = 3). ^##^*p* < 0.01, ^###^*p* < 0.001 vs. Sham group. ^*^p < 0.05, ^**^p < 0.01 vs. MCAO group. For SOD, one-way ANOVA, *F* (3, 8) = 38.94, *p* < 0.0001. For MDA, one-way ANOVA, *F* (3, 8) = 10.22, *p* = 0.0041. For GSH/GSSG, one-way ANOVA, *F* (3, 8) = 7.13, *p* = 0.0119. For DHE in cortex, one-way ANOVA, *F* (3, 8) = 11.63, *p* = 0.0027. For DHE in striatum, one-way ANOVA, *F* (3, 8) = 19.57, *p* = 0.0005.

SOD is a family of enzymes that catalyze the dismutation of the superoxide anion very efficiently. GSH plays a critical role in guarding cells against oxidative damage. They are crucial to maintaining redox homeostasis. MDA is the metabolite of lipid peroxidation^25^. After administration of CZK, the activity of SOD and GSH content in rat brains increased significantly, but the content of MDA had no significant change (Fig. 4A–C). A large amount of ROS was produced and accumulated in the ischemic penumbra of the cortex and striatum of MCAO rats, which led to the aggravation of brain injury. After administration with CZK, ROS levels in both two regions decreased significantly (Fig. 4D,E). It showed that CZK can not only reduce the content of ROS but also promote the activity of antioxidant enzymes.

### CZK alleviated neuronal damage and oxidative DNA injury

**Figure 5:**
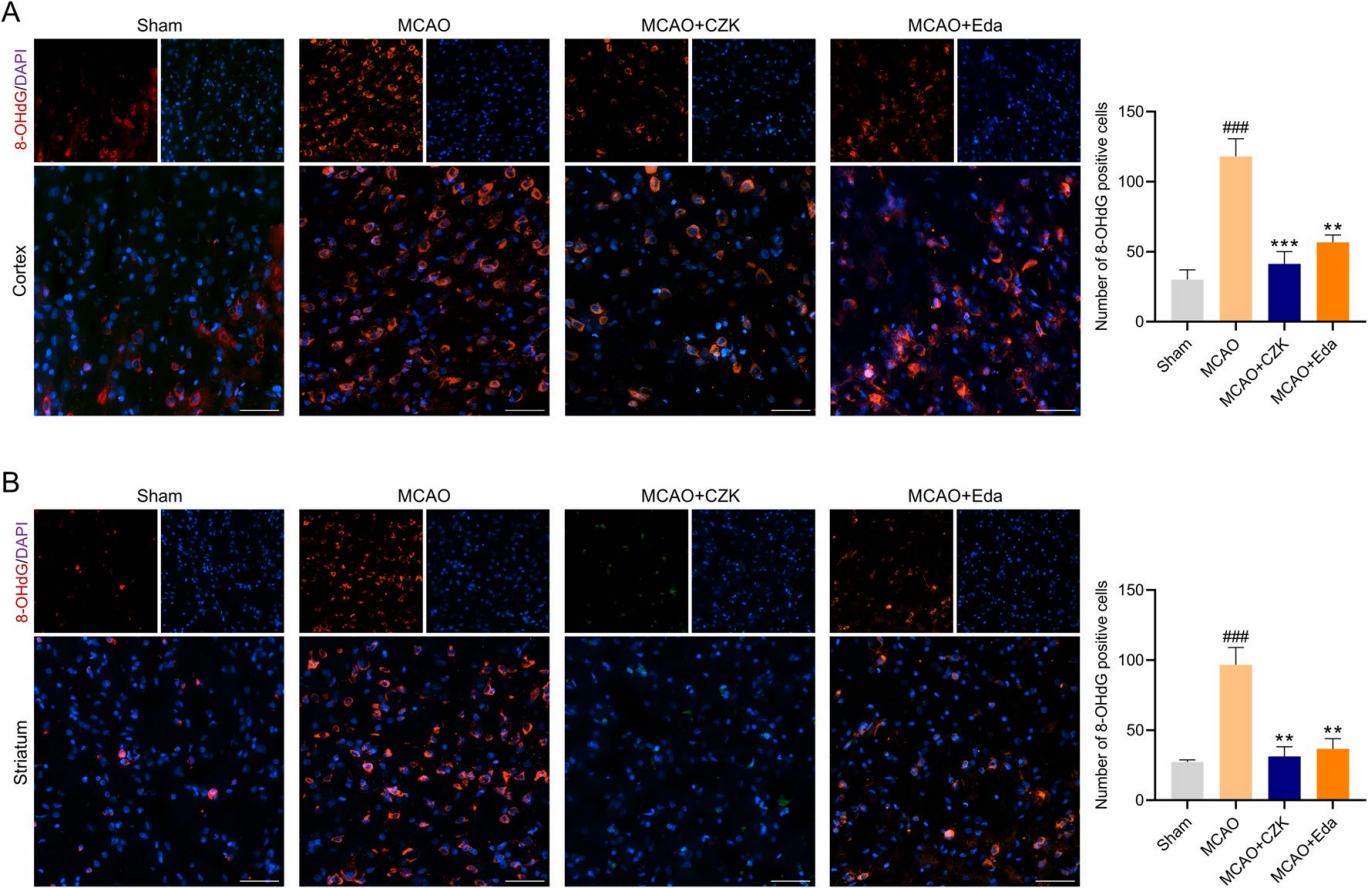
Effect of CZK on oxidative DNA damage in ischemic penumbra at 24 h after MCAO. (**A**) Representative microphotograph in the cortex region and quantification analysis of 8-OHdG-positive cells. (**B**) Representative microphotograph in the striatum region and quantification analysis of 8-OHdG-positive cells. Scale bar = 50 μm. Data were presented as the mean ± SEM (*n* = 3). ^###^*p* < 0.001 vs. Sham group. ^**^p < 0.01, ^***^p < 0.001 vs. MCAO group. For cortex, one-way ANOVA, *F* (3, 8) = 19.62, *p* = 0.0005. For striatum, one-way ANOVA, *F* (3, 8) = 16.80, *p* = 0.0008.

Oxidative stress is one of the pathological mechanisms of neuronal damage. When exposed to high ROS levels, guanines on DNA were attacked by ROS to form 8-OHdG, which was regarded as a biomarker of DNA oxidation^26^. The number of 8-OHdG positive cells in the ischemic penumbra of MCAO groups increased compared with the sham operation group, and the number decreased significantly after CZK administration (Fig. 5).

**Figure 6:**
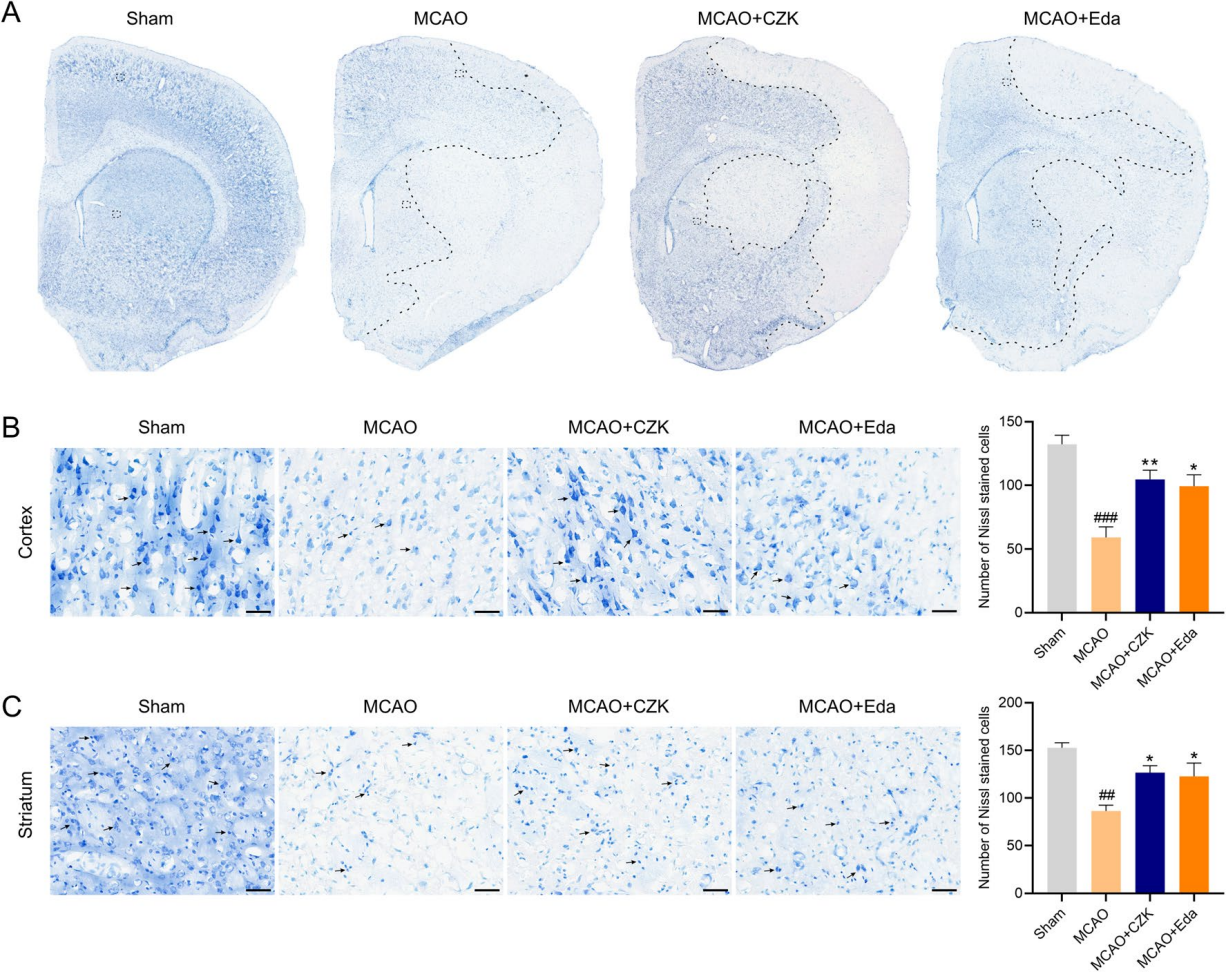
CZK improved neuronal damage in ischemic penumbra at 24 h after MCAO. (**A**) A schematic elucidation of Nissl staining in the right brain. Dotted line indicated the ischemic penumbra. Black boxes represent the observed areas of brain slices. (**B**) Representative microphotograph in the cortex region and quantification analysis of Nissl-stained cells. (**C**) Representative microphotograph in the striatum region and quantification analysis of Nissl-stained cells. Scale bar = 50 μm. Data were presented as the mean ± SEM (*n* = 3). ^##^*p* < 0.01, ^###^*p* < 0.001 vs. Sham group. ^*^p < 0.05, ^**^p < 0.01 vs. MCAO group. For cortex, one-way ANOVA, *F* (3, 8) = 14.48, *p* = 0.0013. For striatum, one-way ANOVA, *F* (3, 8) = 9.396, *p* = 0.0053.

Neuronal damage is also one of the pathological factors of ischemic stroke. Nissl staining was used to indicate the damage condition of the neuron after ischemia–reperfusion. The neuron in the cortex and striatum regions were arranged tidily and the cell morphology was intact in the sham group. However, in the MCAO group, there was a large number of damaged neurons in both two regions, and the number of Nissl-stained cells was significantly decreased compared with the sham group. While CZK administration could increase the number of Nissl-stained cells, and the morphology of the neurons were improved (Fig. 6). These results suggested that CZK could alleviate oxidative DNA injury and neuronal damage after ischemic stroke.

### Molecular docking of CZK and Nrf2

In physiological conditions, Nrf2 binds to the substrate connexin Keap1 in the cytoplasm and then forms the E3 ubiquitin ligase complex, which makes Nrf2 ubiquitinated and degraded. The kelch domain of Keap1 is the main site of the binding of Keap1 and Nrf2. It is considered to be an effective way to activate Nrf2 by combining it with the kelch domain to inhibit the interaction of Keap1-Nrf2^27^.

**Figure 7:**
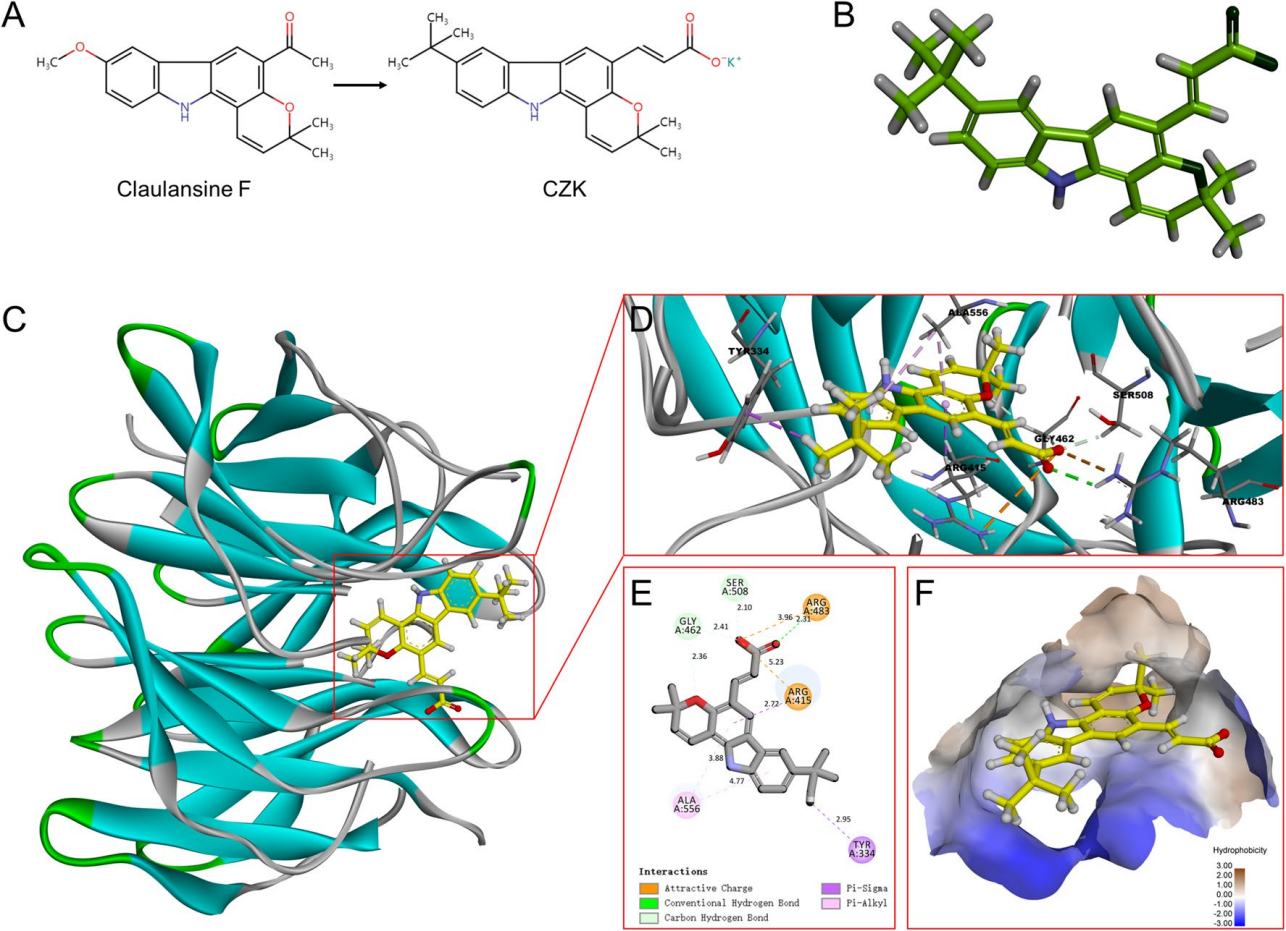
CZK activates Nrf2 by interfering with the interaction between Nrf2 and Keap1. (**A**) The 2D structure of Claulansine F and CZK. (**B**) The 3D structure of CZK. (**C**) Diagram of the binding simulation structure of CZK in the kelch domain of human KEAP1 protein. (**D**,**E**) Solid figure and ichnography of residues interacting with the kelch domain and CZK. (**F**) Hydrophobic interaction of the binding site. The Hydrophobic surface was colored in brown. The figure was created by Discovery Studio v16.1.0 software (https://www.3ds.com/products-services/biovia/products/molecular-modeling-simulation/biovia-discovery-studio/).

In the previous study, we found that Claulansine F isolated from *Clausena lansium* had strong free radical scavenging ability and neuroprotective activity^14^. CZK is a derivative of Claulansine F, which altered its structure by removing the aldehyde group to reduce its potential cytotoxicity (Fig. 7A). We used molecular docking to study the possible binding modes of CZK activating Nrf2, and used 2D and 3D diagrams to show the binding sites of CZK to the Kelch domain of Keap1 (Fig. 7C,D). Hydrophobic interaction is crucial in the regulation of biomolecule recognition and affects the binding process between drug molecules and receptors^28^. The results showed that CZK was inserted into a large hydrophobic chamber (Fig. 7F), and formed a hydrophobic interaction with Ala556 residues (3.88 Å, 4.77 Å). The oxygen on the carboxyl group interacts with Arg483 residues to form hydrogen bonds (2.31 Å). In addition, CZK and kelch domain could form Pi-Sigma, carbon-hydrogen bond, and attractive charge, and four potential active sites (Arg415, Gly462, Ser508, Try334) were included (Fig. 7E). In short, CZK may bind to Keap1 and further dissociate Keap1-Nrf2 combination. This procedure promotes the release of Nrf2 and induces its nuclear translocation, which activates antioxidant elements.

### CZK improves Nrf2 and its target-antioxidative proteins

**Figure 8:**
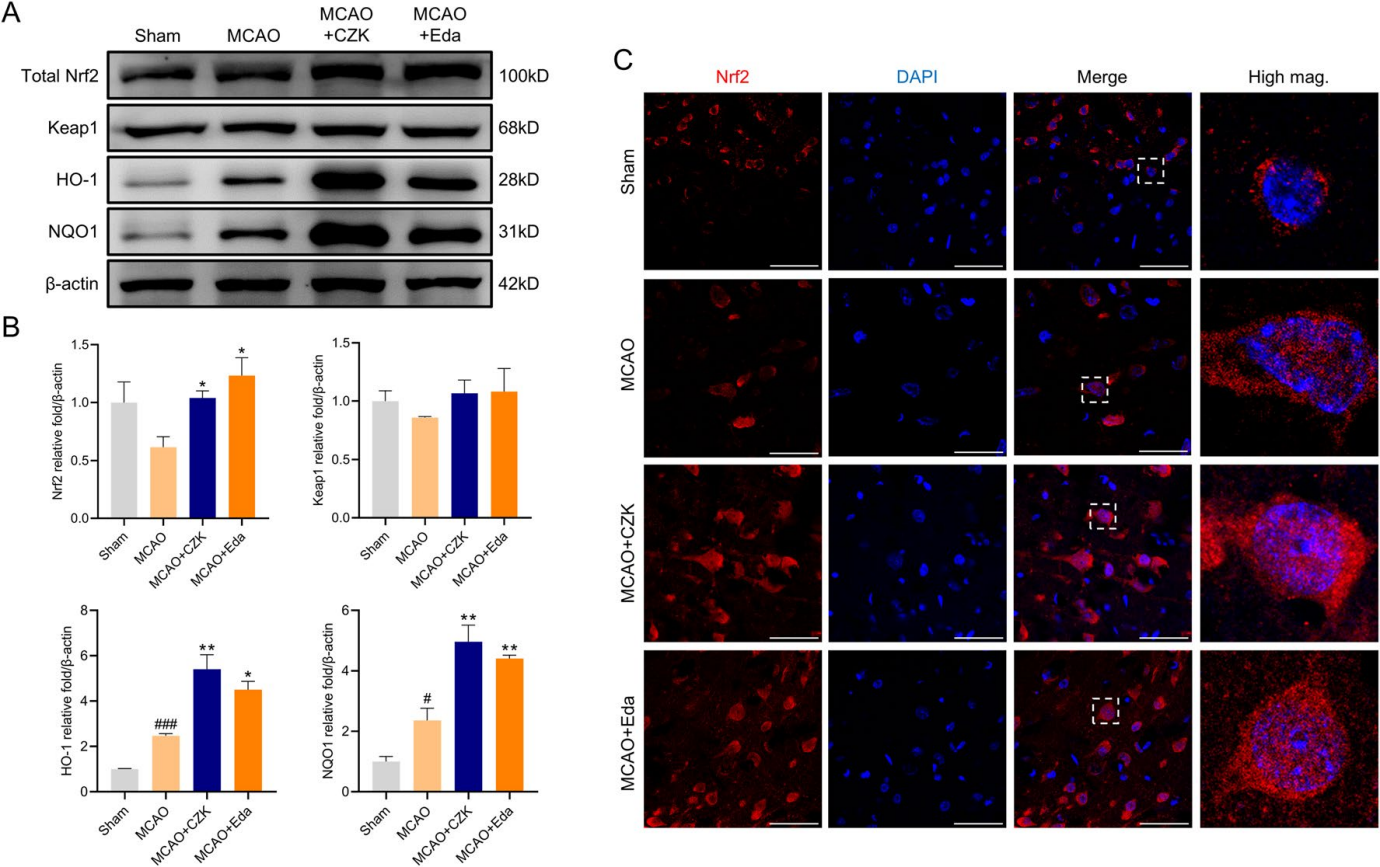
CZK activated Nrf2 signaling and up-regulated antioxidant enzymes expression. (**A**,**B**) Representative western blot image and quantitative analysis of the total protein expression treated by CZK. (**C**) The effect of CZK on nuclear translocation of Nrf2 in the cortex was determined by immunofluorescent assay. Scale bar = 50 μm. Data were presented as the mean ± SEM (*n* = 3). ^#^*p* < 0.05, ^###^*p* < 0.001 vs. Sham group. ^*^p < 0.05, ^**^p < 0.01 vs. MCAO group. For Nrf2, one-way ANOVA, *F* (3, 8) = 3.967, *P* = 0.0529. For Keap1, one-way ANOVA, *F* (3, 8) = 0.6903, *p* = 0.5831. For HO-1, one-way ANOVA, *F* (3, 8) = 28.59, *p* = 0.0001. For NQO1, one-way ANOVA, *F* (3, 8) = 26.90, *p* = 0.0002.

Nrf2 is an important regulatory protein of redox balance in vivo. When cells are under oxidative stress, Nrf2 enters the nucleus and binds to, thus enhancing the expression of downstream antioxidases and promoting the scavenging of free radicals and body repair^29^. Hence, we detected the protein expression of Nrf2 and downstream elements, and all the original blots were presented in Supplementary Figs. 1–5. Nrf2 decreased in the peri-infarct region of the MCAO group, while this depressed expression was significantly increased by CZK administration. For the antioxidases like HO-1 and NQO1, MCAO surgery increased their expression in the peri-infarct region, while the protein expression levels in the CZK treatment group were significantly further elevated (Fig. 8A,B). We also detected the protein expression of Keap1, but there was no obvious change. It indicated that CZK did not decrease the expression of Keap1 but break the Keap-Nrf2 structure to release Nrf2. The immunofluorescence staining revealed that CZK enhanced Nrf2 intensity in the ischemic penumbra of the cerebral cortex. And the colocalization of Nrf2 and DAPI was increased, showing that CZK induced Nrf2 nucleus translocation (Fig. 8C).

## Discussion

Previous studies revealed that *Clausena lansium* had an excellent therapeutic effect in the therapy of Parkinson’s disease and Alzheimer’s disease^16,30^. Claulansine F, a carbazole alkaloid isolated from *Clausena lansium*, had a strong neuroprotective activity that could promote neurogenesis, neuronal differentiation, anti-apoptosis, and free radicals scavenging^31–34^. However, toxicity is an integral part of its drug development. The aldehyde groups in Claulansine F structure make it likely to produce potential toxicity. Therefore, CZK was created to reduce the risk of Claulansine F.

The prediction of toxicity indicated that Claulansine F might inhibit the hERG channels and thus produce cardiotoxicity^35^. Moreover, Claulansine F perhaps had carcinogenicity, mutagenicity and AMES toxicity, while CZK did not (Table 1). We observed that CZK had no significant cytotoxicity at a concentration less than 50 μM in the short term. In contrast, Claulansine F showed a tendency to inhibit SH-SY5Y cell viability at 12.5 μM (Fig. 1A). With the increase in incubation time, there was a significant difference in cell viability between the two groups, and the cell viability of CZK group was significantly higher than that of Claulansine F group (Fig. 1B,C). Meanwhile, after reoxygenation of 24 h, CZK could improve OGD/R-induced cell injury at the concentration from 1.5625 to 12.5 μM, while Claulansine F did not (Fig. 1D). The above results suggest that Claulansine F may produce cytotoxicity during long-term administration, and the modified CZK can reduce this risk without losing its neuroprotective activity.

It was found that CZK alleviated the brain injury of MCAO rats in a dose-dependent manner from 10 to 50 mg/kg (Fig. 3B), as revealed by decreased infarct ratio, edema ratio, and neurological deficit scores (Fig. 3C–E). We found that 50 mg/kg CZK exhibited the best therapeutic effect, and was superior to Edaravone. Therefore, CZK at the concentration of 50 mg/kg was used for further investigation for treating ischemic stroke.

Oxidative stress could exacerbate cerebral lesions during reperfusion. Reducing the production of ROS may decrease the risk of oxidative stress^36^. As mentioned, Claulansine F and another derivative CZ-7 had an extraordinary effect on decreasing ROS production. Therefore, we also detect the free radical scavenging ability of CZK. For hydroxyl radical, the IC_50_ of Edaravone was equal to 790 nM^14^. And in this study, the IC_50_ of CZK was equal to 77.08 nM (Fig. 2), which was much stronger than that of Edaravone. Additionally, CZK treatment enhanced the activities of SOD and GSH, indicating the improvement of antioxidant system (Fig. 4A–C). Generally, excessive ROS often followed by DNA damage. 8-OHdG is an acknowledged biomarker of oxidative DNA damage. We also found that ROS levels were reduced in the ischemic penumbra of the cortex and striatum (Fig. 4D,E), and the number of 8-OHdG positive cells decreased significantly by CZK (Fig. 5). Neuronal injury is one of the pathological features of ischemic stroke. On the other hand, the neuronal damage caused by oxidative stress cannot be ignored. CZK treatment effectively improved the morphology of neurons in the ischemic penumbra and reduce the disintegration of Nissl bodies (Fig. 6). These results further suggested that CZK could improve oxidative stress injury after ischemic stroke.

Nrf2, encoded by the NFE2L2 gene, is a stress-responsive transcription factor closely related to redox equilibrium. It controls the expression of antioxidant and detoxification genes such as SOD, HO-1, NQO1, and Glutathione S-transferase^37^. Under the physiological conditions, the Neh2 domain of Nrf2 combines with the Kelch domain of Keap1 and its activity was repressed by Keap1 and facilitates Nrf2 ubiquitination^38^. However, under pathological conditions stimulated by ROS, Nrf2 is liberated from Keap1-mediated repression. Then Nrf2 can translocate into the nucleus directly and act as a regulatory transcription factor^39^. Hence, we selected the Kelch domain of Keap1-Nrf2 as the targeted receptor in molecular docking. The docking results indicated that CZK might interact with Keap1-Nrf2 Kelch domain effectively, demonstrating that CZK could treat ischemic stroke through Nrf2 pathway (Fig. 7). Consequently, we verified our findings in experimental research. The protein expression levels of Nrf2, HO-1, and NQO1 were up-regulated and the colocalization of Nrf2 and the nucleus were increased after CZK treatment (Fig. 8). In summary, CZK can not only clear the excessive ROS and reduce DNA damage in the ischemic penumbra but also improve the Nrf2-mediated antioxidant capacity, maintain the redox balance, and prevent the further expansion of oxidative damage.

## Conclusion

Our present work revealed that CZK, a derivative of Claulansine F from *Clausena lansium*, could eliminate the cytotoxicity of Claulansine F without losing its neuroprotective activity. And CZK could alleviate cerebral infraction and edema volume in the ischemic brain. In addition, CZK can inhibit excessive ROS and enhance antioxidant capacity, the underlying mechanism may involve the activation of Nrf2 pathway.

## Supplementary Information

Supplementary Figures.

## Data Availability

The datasets used and analyzed during the current study available from the corresponding author on reasonable request.

## Abbreviations

8-OHdG: 8-Hydroxy-2’-deoxyguanosine
ARE: Antioxidant response element
BSA: Bovine serum albumin
CCA: Common carotid artery
DHE: Dihydroethidium
DMEM: Dulbecco’s modified eagle medium
ECA: External carotid artery
EPR: Electron paramagnetic resonance
GSH: Reduced glutathione
GSSG: Oxidized glutathione disulfide
HO-1: Heme oxygenase-1
ICA: Internal carotid artery
Keap1: Kelch-like ECH-associated protein 1
MCA: Middle cerebral artery
MCAO: Middle cerebral artery occlusion
MDA: Malondialdehyde
NQO1: NAD(P)H quinone oxidoreductase 1
Nrf2: Nuclear factor erythroid 2-related factor 2
OGD/R: Oxygen–glucose deprivation/reoxygenation
PBS: Phosphate buffered saline
PDB: RCSB protein data bank
ROS: Reactive oxygen species
rt-PA: Recombinant tissue plasminogen activator
SOD: Superoxide dismutase
TTC: 2,3,5-Triphenyltetrazolium-chloride
