# Supplementary material for: CZK, a novel alkaloid derivative from Clausena lansium, alleviates ischemic stroke injury through Nrf2-mediated antioxidant effects
Source: Sci Rep. 2023 Apr 13;13:6053. doi: 10.1038/s41598-023-32999-2 (PMC10101984; doi:10.1038/s41598-023-32999-2)
Supplement: Supplementary file 1 — Supplementary Figures. [file 41598_2023_32999_MOESM1_ESM.docx]

**Supplementary information-Immunoblots**


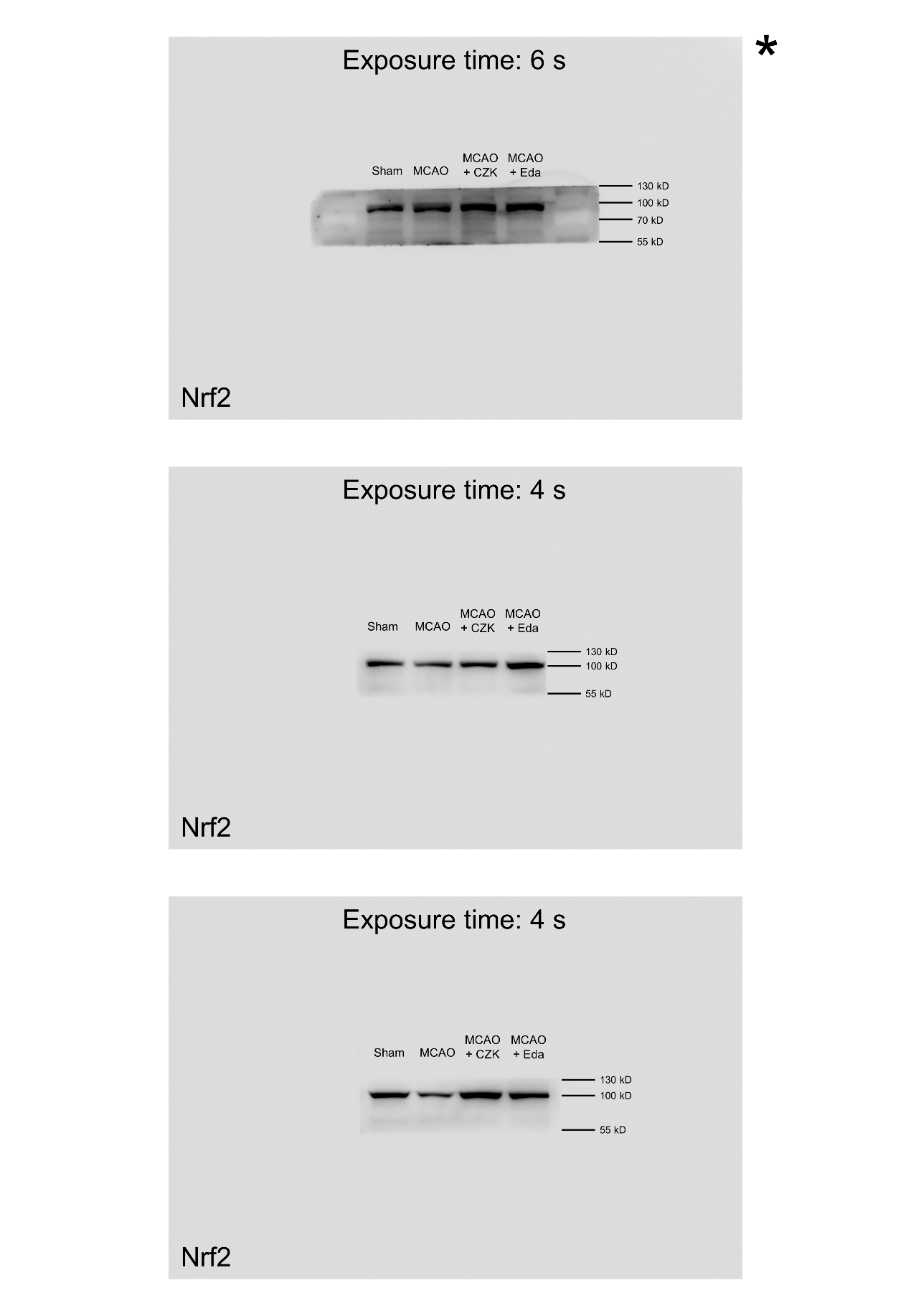


**Supplementary Figure 1.** All original immunoblots used for statistics relating to Nrf2. “*****” were represented as the original image corresponding to Figure 8A.


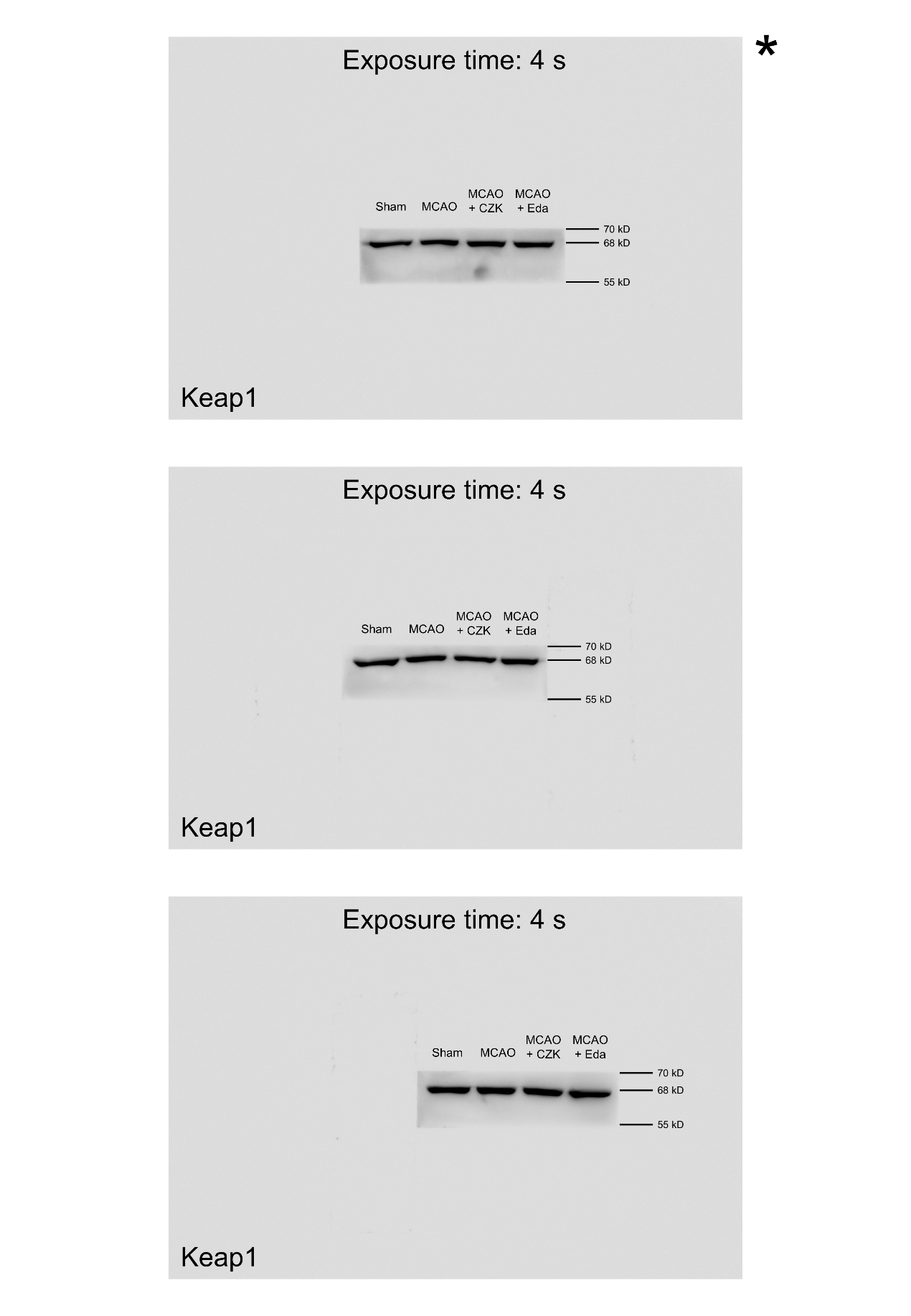


**Supplementary Figure 2.** All original immunoblots used for statistics relating to Keap1. “*****” were represented as the original image corresponding to Figure 8A.


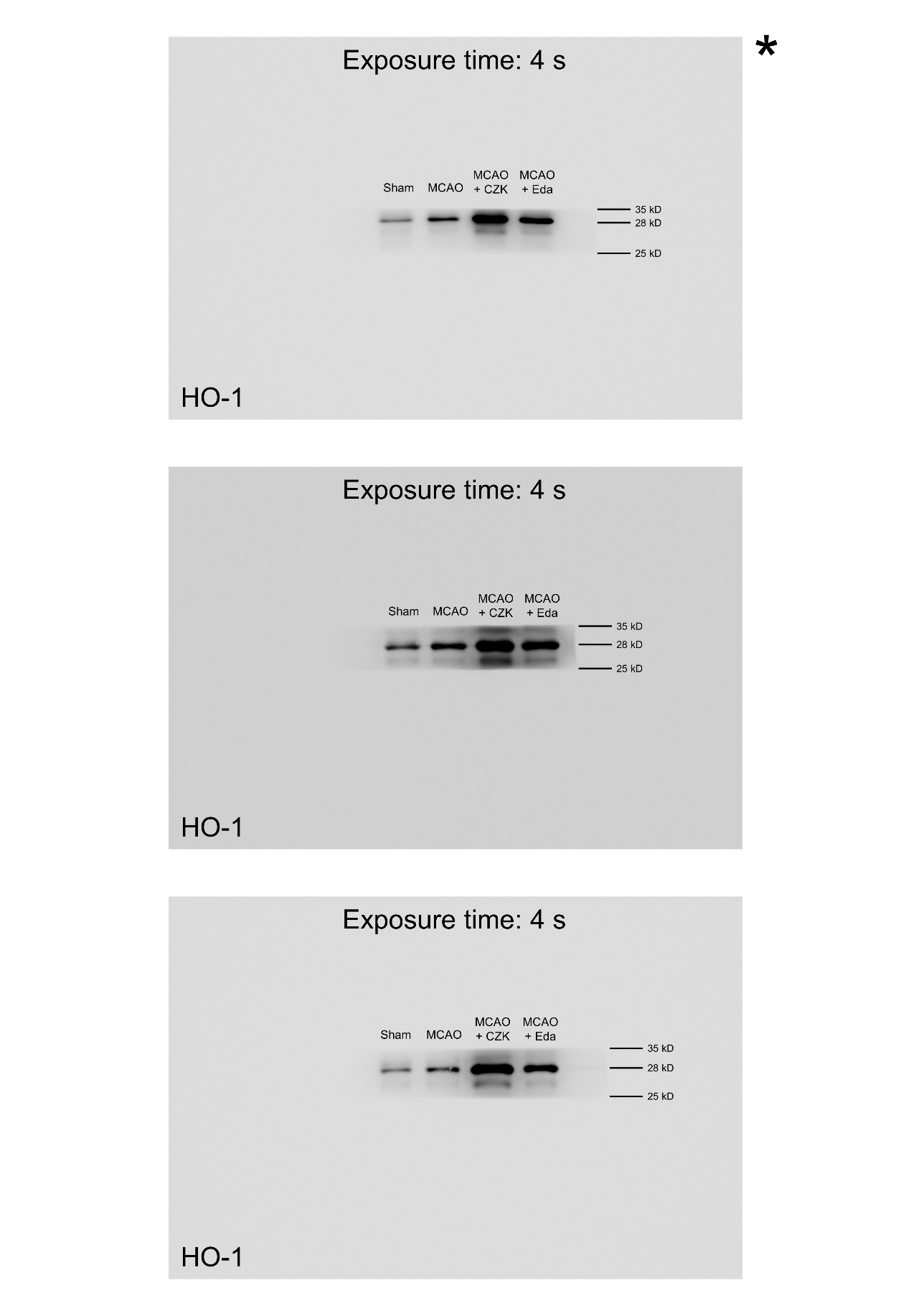


**Supplementary Figure 3.** All original immunoblots used for statistics relating to HO-1. “*****” were represented as the original image corresponding to Figure 8A.


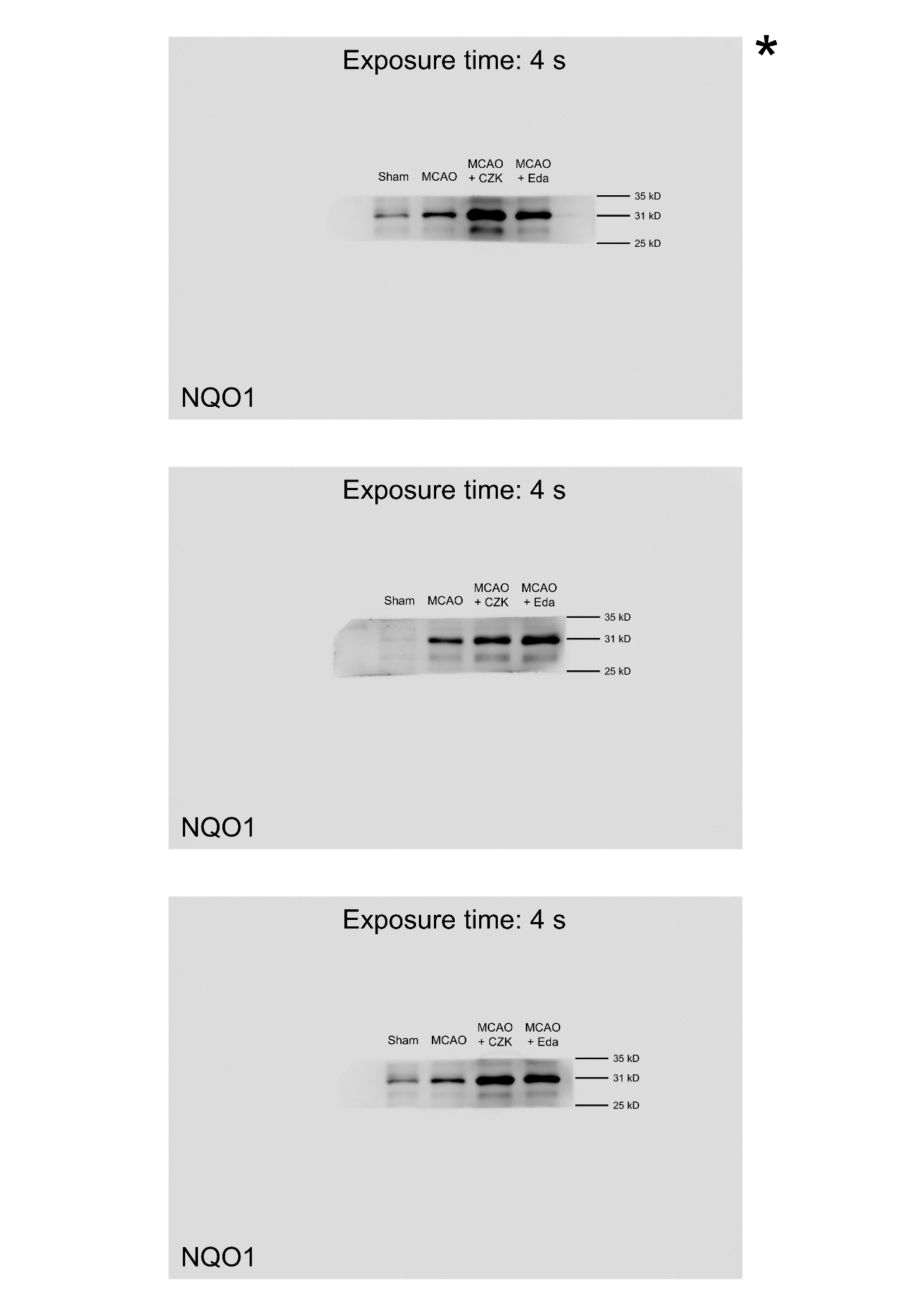


**Supplementary Figure 4.** All original immunoblots used for statistics relating to NQO1. “*****” were represented as the original image corresponding to Figure 8A.


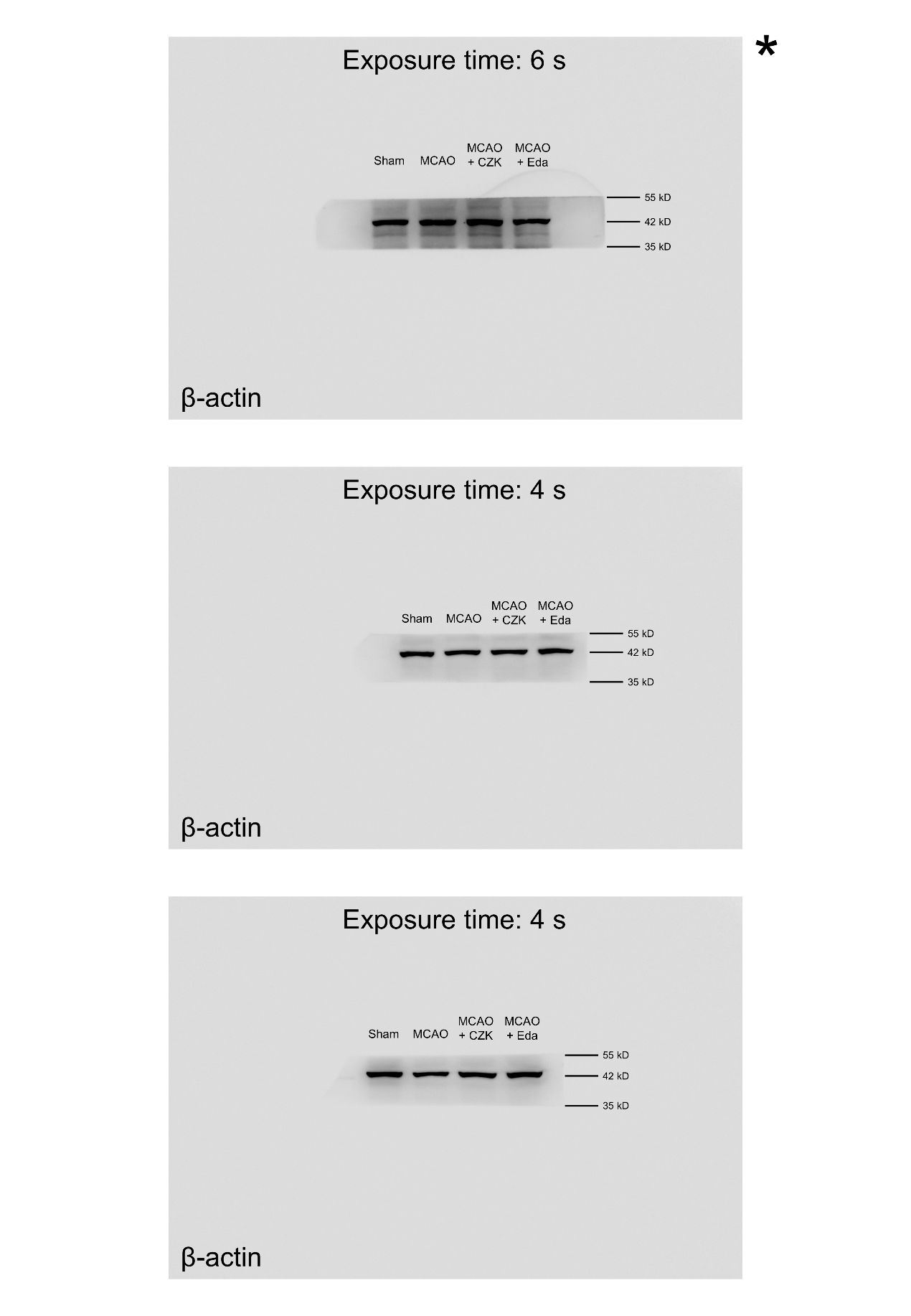


**Supplementary Figure 5.** All original immunoblots used for statistics relating to β-actin. “*****” were represented as the original image corresponding to Figure 8A.
